# Structured Floral Arrangement Program Benefits in Patients With Neurocognitive Disorder

**DOI:** 10.3389/fpsyg.2018.01328

**Published:** 2018-08-03

**Authors:** Hiroko Mochizuki-Kawai, Izumi Kotani, Satoshi Mochizuki, Yuriko Yamakawa

**Affiliations:** ^1^Institute of Vegetable and Floriculture Science, National Agriculture and Food Research Organization, Tsukuba, Japan; ^2^Graduate School of Humanities and Social Sciences, University of Tsukuba, Tsukuba, Japan; ^3^Faculty of Human Sciences, University of Tsukuba, Tsukuba, Japan; ^4^Ibaraki Prefectural University of Health Sciences Hospital, Ami, Japan

**Keywords:** cognitive intervention, floral arrangement, horticultural therapy, visuospatial memory, motivation, neurocognitive disorder, visuospatial ability

## Abstract

We attempted to clarify positive benefits in cognitive abilities and motivation during our cognitive intervention [structured floral arrangement (SFA) program] for patients with neurocognitive disorder due to stroke, traumatic brain injury (TBI), and other related disorders. In this SFA program, participants are required to arrange cut flowers and leaves on absorbent foam according to an instruction sheet. In a previous study of patients with schizophrenia, our SFA program encouraged participants and contributed to stimulating their visuospatial process and memory. Here, 27 patients with neurocognitive disorders participated in this study. Sixteen patients were assigned to an SFA-treated group and participated in six sessions during two phases plus to daily activities. Eleven non-treated patients engaged only daily activities during the same period. We compared Apathy Scale scores and neuropsychological scores between the SFA-treated and non-treated patients. Their mean attendance rate was more than 90% during the two phases. SFA-treated patients copied a Rey–Osterrieth complex figure more accurately than non-treated patients (*p* < 0.05) during the later intervention phase, whereas during the earlier phase, accuracy was comparable between treated and non-treated groups. In the SFA-treated group, recall scores also improved (*p* < 0.01), and the positive outcomes were maintained for about 3 months (*p* < 0.05). The Apathy Scale scores did not show significant change in either the SFA-treated or non-treated groups. Our present results suggest that the SFA program encouraged continuous participation to cognitive intervention and was useful for ameliorating dysfunctions in visuospatial memory and recognition in patients with neurocognitive disorder.

## Introduction

Various cognitive interventions have been developed to improve cognitive functions of patients with neurocognitive disorder due to stroke, traumatic brain injury (TBI), Alzheimer’s disease, and other-related disorders (van de Ven et al., 2001; Talassi et al., 2007; De Vreese et al., 2008; Mapelli et al., 2013; Zucchella et al., 2014; Hallock et al., 2016; Panerai et al., 2016; Orrell et al., 2017; Pérez-Martín et al., 2017). For patients with acute ischemic or hemorrhagic stroke, Zucchella et al. (2014) conducted cognitive training (CT) in which patients performed computer-based exercises (e.g., time orientation, word search puzzles, searching for targets among distractors, and calculation) for 16 h during 4 weeks. Participants demonstrated considerable improvement in domains of visual attention and verbal memory. CT generally consists of computer-based or paper-and-pencil cognitive exercises for targeting specific functions, including memory, attention, and executive function (Talassi et al., 2007; Panerai et al., 2016), and CT has shown significant cognitive outcomes in patients with stroke (Zucchella et al., 2014), TBI (Hallock et al., 2016), and multiple sclerosis (Pérez-Martín et al., 2017).

Cognitive stimulation therapy (CST) is also a well-established cognitive intervention for elderly people with neurocognitive disorder. Participants engage in reality orientation and other enjoyable activities such as word games, being creative, and sound recognition (Spector et al., 2003; Mapelli et al., 2013; Panerai et al., 2016; Orrell et al., 2017). General cognitive function and behavioral symptoms were improved through daily CST treatments in patients with elderly dementia (Spector et al., 2003; Mapelli et al., 2013). Panerai et al. (2016) proposed a new combined CT and CST treatment program (intensive cognitive activation), designed according to each participant’s neurocognitive dysfunction. CT seems relatively successful for people with mild neurocognitive disorder; CST seems more acceptable for people with severe neurocognitive disorder, including elderly dementia (Buschert et al., 2010; Panerai et al., 2016). When treated with an appropriate program, patients experienced positive outcomes in neurocognitive functions (Buschert et al., 2010; Panerai et al., 2016).

In addition to program content, continuous participation to cognitive intervention appears important in achieving significant outcomes (Orrell et al., 2017). In home-based CST programs, positive effect on cognition and quality of life (QOL) were not found in people with dementia (Orrell et al., 2017) even though other studies had revealed benefits (Spector et al., 2003; Knapp et al., 2006; Buschert et al., 2010; Mapelli et al., 2013). Orrell et al. (2017) cited low levels of adherence to the intervention as a potential limitation of their study. For inducing maximum cognitive outcomes, proposing an attractive intervention program with continuous participation may be important. Recently, acceptable and attractive methods have been proposed for cognitive improvement. Participants with mild cognitive impairment and Alzheimer’s disease reported high satisfaction and motivation after completing a 4-week serious game as CT (Manera et al., 2015).

We have developed a structured floral arrangement (SFA) program, combining benefits of CT and horticultural therapy, in which participants were required to arrange cut flowers and leaves (foliages) on absorbent foam according to an instruction sheet. The SFA treatment program contributed to stimulating abilities of visuospatial process and spatial memory like CT, and it also encouraged continuous participation (Mochizuki-Kawai et al., 2010). In patients with schizophrenia, the SFA participation rate nearly doubled that of other daily activities (e.g., singing, cooking, light exercise) (Yamakawa et al., 2008; Mochizuki-Kawai et al., 2010). This program’s acceptability has elements in common with horticultural therapy, which is useful for improving psychiatric symptoms as well as for motivating participants by using natural materials (Lee and Kim, 2008; Pereira and Pereira, 2009).

As training for visuospatial ability, the SFA procedure resembles that of a pegboard (Burdea, 2003; Ashman et al., 2008) or block design (Ben-Yishay, 1974; Young et al., 1983) and appears to include more complex manipulation of three-dimensional visual information with high load on visuospatial memory (Mochizuki-Kawai et al., 2010). In a case study with chronic TBI, a symptom of unilateral spatial neglect, and a deficit of visuospatial recognition, improvement was shown through SFA treatments, and remedial effects were identified 5 months after treatment (Mochizuki-Kawai et al., 2013). Furthermore, a chronic stroke patient showed improvements in scores on Rey–Osterrieth complex figure copy tests through SFA treatments (Mochizuki-Kawai, 2016). SFA would predictably be effective for ameliorating visuospatial dysfunction although sufficient trials with multiple cases have not yet provided empirical evidence.

To clarify cognitive outcomes, the present study investigated change using group comparisons of visuospatial abilities with SFA treatments. We also attempted to clarify SFA treatments’ horticultural effects. For one group, real flowers, and leaves were used as materials and, for the other, colored sticks; then cognitive outcomes and motivation for the program were compared between groups. We believe that the present study of SFA contributes to further development of cognitive interventions for people with neurocognitive disorder.

## Materials and Methods

### Participants

We recruited patients with neurocognitive disorders from 2 day-care facilities whom directors agreed to cooperate on our study. In one facility, the number of daily users was from 35 to 50, and about 20 in the other one. These users were suffered from neurocognitive dysfunctions. They engaged daily in activities including light exercise, fancywork, and horticultural work, were able to communicate with easy oral language, and were physically capable of light work. We introduced our research to all users older than 18 years who had no congenital cognitive dysfunctions in the two facilities, and recruited 27 patients who agreed to participate our research. The 27 patients were randomized into two groups (SFA treated = 16, non-treated = 11) nearly equal in age, years of education, illness, and MMSE score (**Table 1**). The number of SFA-treated patients was about 1.5 times larger than the number of non-treated Control group, because the 16 SFA-treated patients subdivided into two subgroups.

**Table 1:** Characteristics of SFA and Control groups.

	SFA (*n* = 16)	Control (*n* = 11)
Sex (m/f)	13/3	10/1
Etiology TBI	8	6
Stroke	4	4
Others	4	1
Mean age	43.8 (10.4)	40.6 (12.3)
Mean years of education	12.9 (2.4)	12.1 (0.3)
Mean years since illness	5.13 (5.6)	6.99 (5.6)
Mean MMSE score	24.4 (4.7)	23.4 (7.2)

*SFA, structured floral arrangement program treatment; TBI, traumatic brain injury; MMSE, Mini-Mental State Examination (Folstein et al., 1975). Standard deviations are shown in parenthesis.*

After assignment to the SFA-treated group, 16 patients participated in the SFA treatments in addition to the daily program. They showed mild to moderate neurocognitive deficits due to TBI, stroke, or other diseases (e.g., herpes encephalitis, multiple sclerosis). In the Control group, 11 patients showed mild to moderate neurocognitive dysfunctions because of TBI, stroke, or herpes encephalitis. Mean scores of the Mini-Mental State Examination (MMSE) were 24.4 in the SFA and 23.4 in the Control group; about 40% of all same were recognized as having dementia according to the MMSE cut-off score (Folstein et al., 1975). No significant differences were observed between patients in the SFA and Control groups in their characteristics (**Table 1**).

This study was approved by the Ethics Committees of the University of Ibaraki Prefectural University of Health Sciences Hospital. We obtained written informed consent from all adult participants and from the parents of non-adult participants before participating in the study. When participants could not sign due to their brain dysfunction, we obtained written informed consent from their family members after confirming participants’ agreements.

### Procedure

**FIGURE 1:**
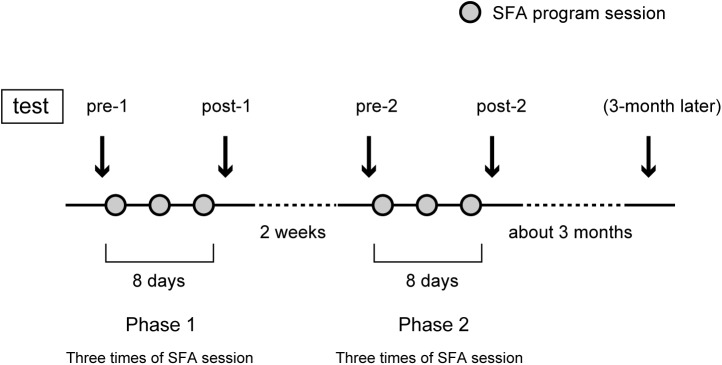
Experiment schedule of the SFA-treated group.

**FIGURE 2:**
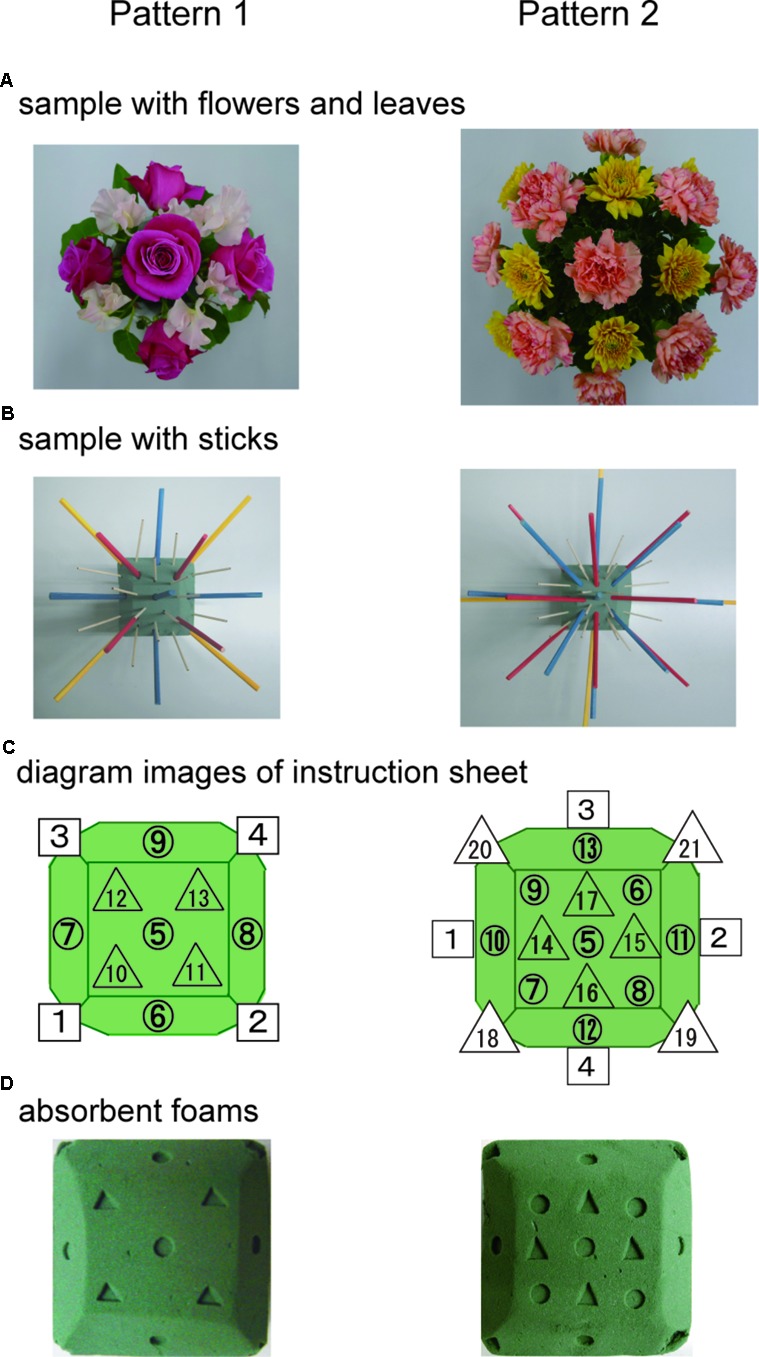
Floral arrangement samples with flowers and leaves **(A)** and samples with colored sticks **(B)**. Diagram images of base foam shown in a instruction sheet **(C)**, and the foams **(D)** used in the SFA program.

**Figure 1** shows the SFA group’s experimental schedule, that is, two treatment phases at intervals of 2 weeks. In each phase, three sessions of SFA treatment (30–40 min) were conducted within 8 days. Neuropsychological tests were examined before and after each phase (**Figure 1**: pre-1, post-1, pre-2, and post-2). The 16 SFA patients were divided randomly into two similar subgroups that did not differ in age, years of education, time since onset of illness, and MMSE scores. The first subgroup, flower–stick SFA patients (*n* = 7) participated with real flowers and leaves (**Figure 2A**) in phase 1, and, in phase 2, they used colored sticks instead (**Figure 2B**). The second subgroup, the stick–flower SFA patients (*n* = 9) first used sticks and then real flowers and leaves, in reverse order from the first group. Both groups’ total treatment times were the same. We prepared two floral arrangement patterns with differing levels of difficulty (**Figure 2**, Patterns 1 and 2). Patients were instructed to make the easier pattern (**Figure 2**, Pattern 1) on the first day of each phase and the more complex pattern (**Figure 2**, Pattern 2) on the latter 2 days.

### SFA Program Treatment

Structured floral arrangement program treatments were conducted in a group setting according to the procedure described in Mochizuki-Kawai et al. (2010). In a session, participants made the same designed arrangement twice on absorbent foam. For the first one, they followed step-by-step instructions provided by staff members (neuropsychologist, occupational therapist, or psychiatrist), based on an instruction sheet. Diagrams of the impressed foam were shown on the instruction sheet (**Figure 2C**), which guided the order and location to place each material on the foam (**Figure 2D**). For example, as a fifth procedure of Pattern 1, a pink rose was to be placed vertically on the foam’s No. 5 center circle impression (**Figure 2** left).

During the treatment’s second half, patients were instructed to complete the arrangement by referring to the instruction sheet without any staff assistance. To evaluate patients’ arrangement skills, we photographed their work and recorded with a stopwatch the time they took to finish the second arrangement on each SFA treatment session. After each SFA treatment with flowers and leaves, patients took their arrangements home with them.

### Cognitive Tests

To clarify cognitive outcomes, we used the Rey–Osterrieth complex figure test (copy and immediate recall version) (Rey, 1941; Osterrieth, 1944) and the digit span and block-tapping tests (Wechsler Memory Scale-Revised, Wechsler, 1987). These assessments were executed four times, before and after the two phases (**Figure 1**: pre-1, post-1, pre-2, and post-2). The Rey–Osterrieth complex figure test was administered only to the SFA-treated patients about 3 months after phase 2 as a follow-up test. Control group patients spent their time as usual at the day-care facility with daily activities and participated only in neuropsychological tests on the same schedule as the SFA group, except for the 3-month follow-up. Because we predicted that Rey scores improved only in the SFA-treated group during two test phases, we did not plan the following test in the Control group. Using the Apathy Scale (Starkstein et al., 1993; Okada et al., 1997) before and after each phase, we also evaluated the level of interest and concern in each group; we recorded their attendance rate to represent their motivation toward the program. We conducted the *t*-test or two-way ANOVA and simple main comparison to clarify the SFA program’s outcomes.

## Results

The SFA program’s mean attendance rates were 91.7% (with flowers and leaves) and 95.8% (with sticks). We observed that almost all SFA-treated patients continued to try to complete their arrangements and were able to finish them alone in the treatment’s second half. Once in the second session, one TBI patient failed to finish an arrangement with flowers and leaves by himself during phase 1, but he was able to finish his work without any assistance the next treatment day.

### Cognitive Outcomes

**FIGURE 3:**
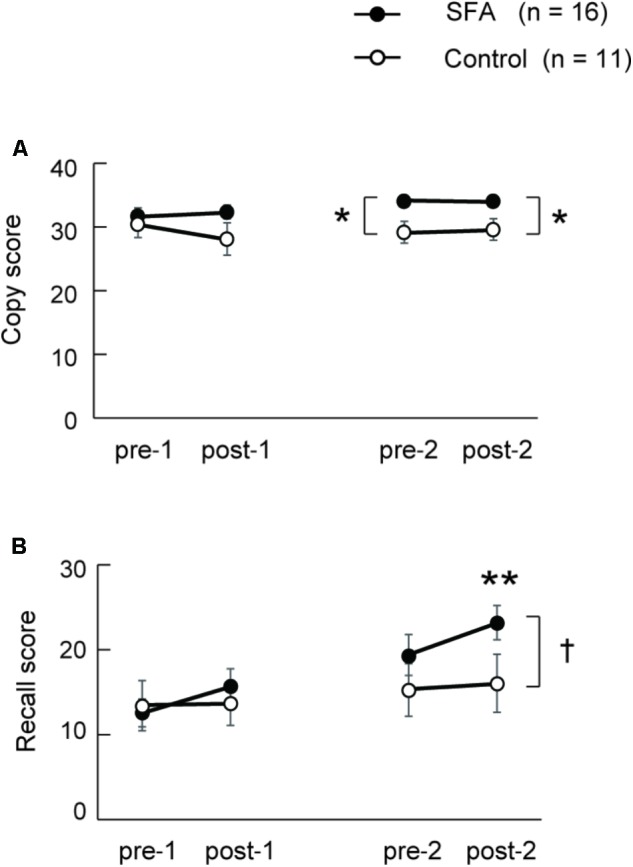
Mean scores of Rey–Osterrieth complex figure copy **(A)** and recall **(B)** test in SAF-treated and non-treated (Control) groups. ^∗^*p* < 0.05, ^∗∗^*p* < 0.01, ^†^*p* < 0.10.

**Figure 3A** shows changes in copy scores on the Rey–Osterrieth complex figure test both in SFA-treated and Control groups during the two phases. We found significant interaction between groups (SFA and Control) and test periods (pre-1, post-1, pre-2, and post-2) (*p* < 0.05). The SFA group’s mean scores were significantly higher than those of the Control group both in pre-2 and post-2; SFA-treated patients could draw the complex figure more correctly than Control patients during phase 2. On recall scores in the Rey–Osterrieth complex figure test, we also detected significant interaction between groups and test periods (**Figure 3B**, *p* < 0.05). The SFA group’s mean scores gradually improved through treatments, and their post-2 mean score was significantly higher than those of the other three tests (*p* < 0.01). We also found a significant trend between the mean scores of the SFA and Control groups in the post-2 (**Figure 3B**, *p* < 0.10).

**FIGURE 4:**
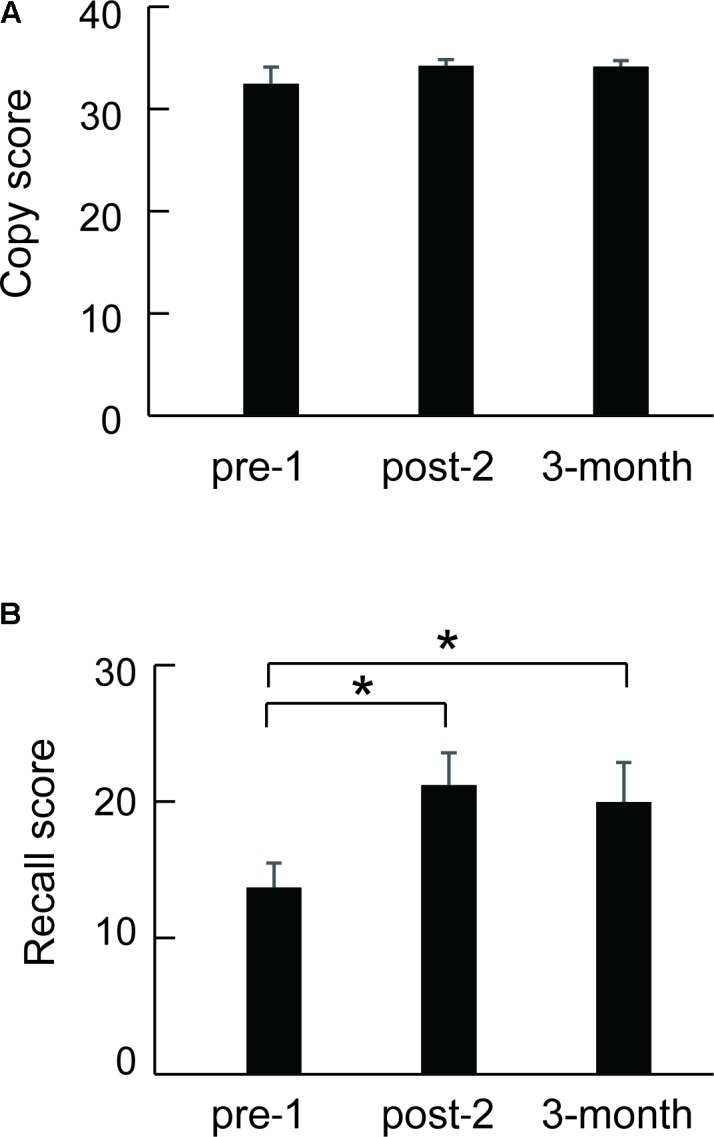
Mean scores of the followed SFA-treated patients (*n* = 11) in the Rey–Osterrieth complex figure copy **(A)** and recall **(B)** test. ^∗^*p* < 0.05.

**FIGURE 5:**
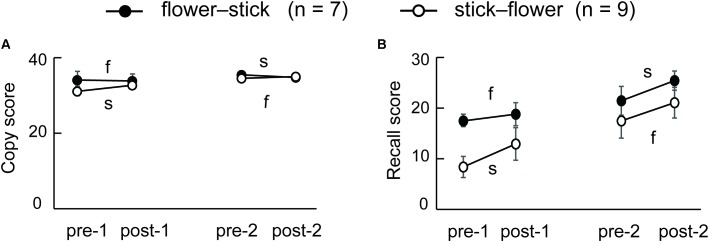
Mean scores of the flower–stick (*n* = 7) and stick–flower (*n* = 9) SFA-treated subgroups in the Rey–Osterrieth complex figure copy **(A)** and recall **(B)** test. f, SFA treatments with flowers and leaves between pre- and post-tests; s, SFA treatments with sticks between pre- and post-tests.

Eleven of 16 SFA patients were followed about 3 months after the final SFA treatment. **Figure 4** shows these patients’ mean Rey–Osterrieth complex scores at each test point (pre-1, post-2, and 3 months later). Copy scores showed no significant differences among test points (**Figure 4A**). By contrast, the mean recall scores at post-2 and after 3 months were significantly higher than those at pre-1 (**Figure 4B**, *p* < 0.05). SFA-treated patients maintained improvement of visuospatial memory for 3 months. We analyzed the Rey–Osterrieth complex data separately between flower–stick (*n* = 7) and stick–flower (*n* = 9) SFA subgroups (**Figure 5**). No significant differences in copy (**Figure 5A**) and recall (**Figure 5B**) scores were found between subgroups. In other words, SFA materials (real flowers and leaves or sticks) did not affect outcomes of the Rey–Osterrieth complex figure test.

**FIGURE 6:**
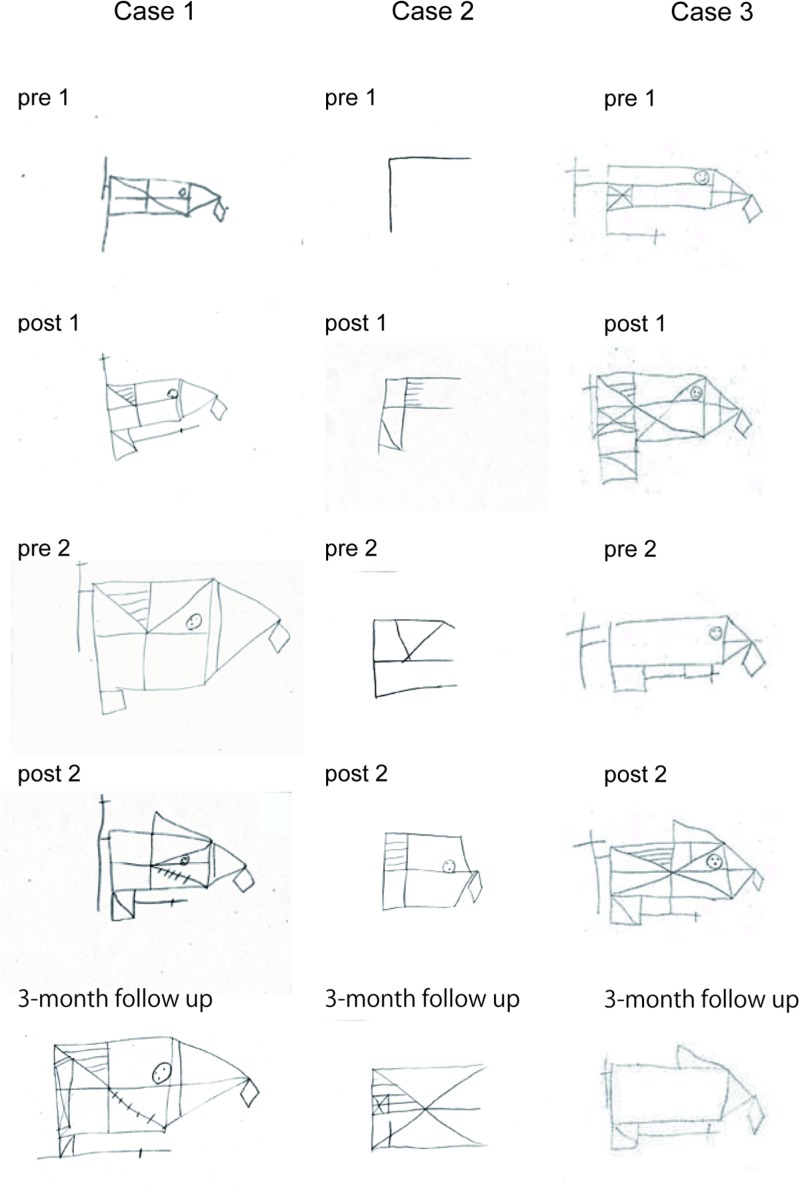
Drawing samples of the Rey–Osterrieth complex figure recall test in three SFA-treated patients.

**Figure 6** shows drawing samples by three SFA-treated patients in the Rey–Osterrieth complex figure recall test. Case 1 was a 24-year-old female (MMSE = 28), who had right-brain lesion due to TBI 4 years before her SFA program participation (flower–stick subgroup). Her performance improved gradually with SFA participation, and she maintained that improvement for 3 months (**Figure 6**, Case 1). Case 2 was a male with mild dementia (MMSE = 22), 48 years old; he suffered from multiple sclerosis onset 2 years before SFA participation (stick–flower subgroup), with bilateral brain damage. Although he performed well on the pre-1 Rey–Osterrieth complex figure copy test (score = 34/36), he revealed severe memory deficit in the recall version (**Figure 6**, Case 2, pre-1). His recall scores improved through SFA treatments, and on the 3-month follow-up test, he appeared to maintain improvement (**Figure 6**, Case 2). Case 3 was a 34-year-old male with moderate cognitive dysfunction (MMSE = 10) due to TBI 2 years before SFA participation (flower–stick subgroup). His copy scores improved from 26.0 (pre-1) to 31.5 (post-2) points during treatment. Although recall scores improved in the two SFA phases, his scores declined after 2-week and 3-month intervals (**Figure 6**, Case 3). Even in older patients, improvements were found in Rey–Osterrieth complex figure recall test. Case 4 (male, 63 years old, MMSE = 22, stroke) recorded 31.5 points in the post-2 test, while he scored 8 points in the pre-1 test. In Case 5 (female, 57 years old, MMSE = 28, TBI), recall scores were increased from 19 to 28 points during SFA interventions. The improvements in recall scores were observed over a wide age range.

**Table 2** shows other tests’ mean scores. Scores on the digit span, block tapping, and the Apathy Scale did not reveal any significant changes in either the SFA or Control patients. From the Apathy Scale, Japanese version, each patient was recognized as having apathy syndrome at over 16 points (Okada et al., 1997). Seven of 16 SFA patients and six of 11 Control patients were recognized as having apathy syndrome in pre-1, and almost all the same patients revealed similar scores in Apathy Scale (Okada et al., 1997) at post-2 evaluation. The remaining nine of 16 SFA-treated non-apathy patients maintained their preferred mental state throughout the experiment, but three of five non-apathy Control patients showed gradually increasing Apathy Scale scores and were recognized as having apathy syndrome at post-2 evaluation.

**Table 2:** Mean cognitive and Apathy test scores in SFA and Control groups.

SFA	Pre-1	Post-1	Pre-2	Post-2
Digit span				
Forward	5.0 (1.7)	5.3 (1.4)	5.6 (1.7)	5.6 (1.5)
Backward	3.2 (1.3)	3.5 (1.7)	3.9 (1.4)	3.8 (1.3)
Block tapping				
Forward	4.9 (0.9)	5.2 (1.3)	4.5 (0.7)	4.9 (1.1)
Backward	4.6 (1.4)	4.2 (0.9)	4.2 (0.8)	4.7 (1.2)
Apathy Scale	13.9 (8.9)	14.6 (8.4)	12.4 (7.0)	14.0 (8.5)

**Control**	**Pre-1**	**Post-1**	**Pre-2**	**Post-2**

Digit span				
Forward	5.3 (1.5)	4.8 (1.8)	5.0 (1.9)	5.1 (1.4)
Backward	2.8 (1.3)	3.0 (1.7)	3.1 (1.4)	3.1 (1.7)
Block tapping				
Forward	5.3 (1.1)	4.5(1.8)	4.5 (1.3)	4.9 (1.3)
Backward	4.5 (1.1)	4.4 (2.2)	4.4 (0.8)	4.4 (1.1)
Apathy Scale	14.2 (6.1)	15.4(6.7)	16.5 (7.8)	17.3 (8.3)

*SFA, structured floral arrangement program treatment.*

### SFA Arranging Work

**FIGURE 7:**
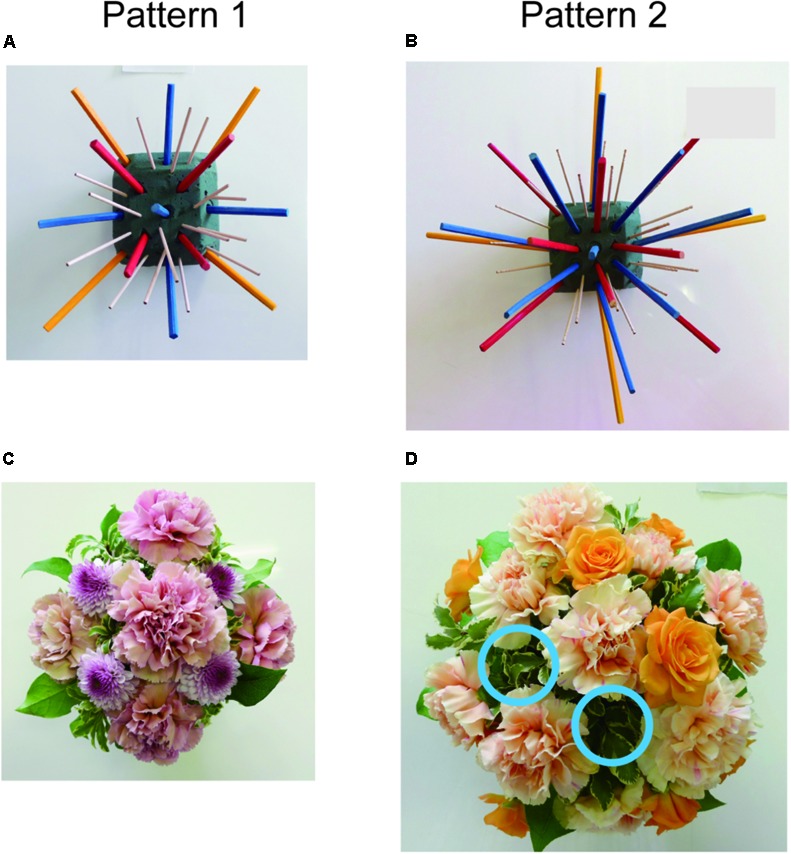
Work samples with sticks **(A,B)** or flowers **(C,D)**, arranged by 35-year-old male without assistance in the SFA program. Blue circles indicate lack of orange roses **(D)**.

Almost all patients could arrange materials correctly in Pattern 1 treatments, but they made mistakes occasionally in Pattern 2, which had a more complex design. **Figure 7** shows samples arranged by a 35-year-old male (TBI, stick–flower SFA subgroup) without any support in the intervention’s second half. Although his copy score was 32.5 points, his recall score (0.5 point) was the worst among SFA group participants in the Rey–Osterrieth complex figure pre-1 test. He finished two arrangement patterns well with sticks, which were all placed correctly on the marked absorbent foam (**Figures 7A,B**). He also arranged flowers and leaves well (**Figure 7C**), but he failed correctly to place two cut flowers (orange roses) in Pattern 2 (**Figure 7D**, blue circle).

**Table 3** shows mean performance times for completing arrangements alone in the SFA treatment’s second half. Mean performance time with flowers and leaves was significantly longer than that with sticks (*p* < 0.01), and participants required more time to finish the arrangement on the second and third days (Pattern 2) than on the first day (Pattern 1) during each phase (*p* < 0.05). As we expected, the Pattern 2 design required a higher load for participants than Pattern 1. In addition, arranging flowers and leaves was more difficult than arranging sticks. We divided performance time by the number of materials (flowers and leaves or sticks) in each program pattern and calculated mean times required for placing each piece of material on the foam’s impression (**Table 4**). Mean times with flowers and leaves were significantly longer than with sticks (*p* < 0.01), and the time did not change within a phase.

**Table 3:** Mean performance times (s) for creating one arrangement with flowers or sticks in the second half of each SFA program.

	Phase 1			Phase 2		
	1	2	3	1	2	3
	***Flower***			***Stick***		
Flower–stick	465	781	743	311	507	522
	(44.8)	(67.4)	(51.3)	(18.6)	(34.4)	(31.8)

	***Stick***			***Flower***		
Stick–flower	179	377	365	361	496	497
	(21.0)	(51.1)	(51.4)	(66.6)	(82.2)	(89.4)

*Flower–stick (n = 7), stick–flower (n = 9).*

**Table 4:** Mean times (s) required for placing each piece of material during the second half of SFA programs.

	Phase 1			Phase 2		
	1	2	3	1	2	3
	***Flower***			***Stick***		
Flower–stick	16.6	19.1	18.1	11.1	12.4	12.7
	(1.6)	(1.6)	(1.3)	(0.7)	(0.8)	(0.8)

	***Stick***			***Flower***		
Stick–flower	6.4	9.2	8.9	12.9	12.1	12.1
	(0.8)	(1.2)	(1.3)	(2.4)	(2.0)	(2.2)

*Flower–stick (n = 7), stick–flower (n = 9).*

## Discussion

We found positive cognitive outcomes with the SFA program in the Rey–Osterrieth complex figure test. SFA-treated patients could draw a complex figure more accurately than Control patients in the later phase. Patients also improved their visuospatial memory through SFA treatments, and positive outcomes were maintained for about 3 months. Even for a patient with severe memory deficits, visuospatial memory scores improved gradually during treatment. Thus, our results revealed that the SFA program enhanced visuospatial analysis, recognition, and memory. These results were consistent with our previous studies’ results (Mochizuki-Kawai et al., 2010, Mochizuki-Kawai, 2016) and results of other studies with CT programs (Zucchella et al., 2014; Hallock et al., 2016; Pérez-Martín et al., 2017). Our present results showed the SFA program useful for ameliorating deficits with visuospatial memory and recognition. SFA has benefits in common with CT programs that efficiently stimulate and improve targeted cognitive functions (Talassi et al., 2007; Panerai et al., 2016). Attendance at this SFA program averaged more than 90%. In contrast, scores of digit span, block tapping, and apathy levels did not change during SFA treatments.

The SFA program’s attendance rate was high, and almost all patients with mild to moderate neuropsychological disorders could finish their arrangements alone during the second half of treatment. The SFA program may be appropriate for a broad range of patients with neurocognitive dysfunctions, and it appears to provide CST-like elements that encourage participation through enjoyable activities (Mapelli et al., 2013; Orrell et al., 2017). In addition, SFA treatments were conducted six times and lasted 30 to 40 min each. Our schedule was relatively briefer than those of other cognitive interventions, during which treatments were conducted from 12 to 40 times, and each session lasted more than 1 h (Mapelli et al., 2013; Zucchella et al., 2014; Panerai et al., 2016; Pérez-Martín et al., 2017). To put it another way, SFA program methods may be adequate for improving visuospatial ability and memory with minimum treatment. These short-term clinical outcomes would likely encourage patients and their family members, and the SFA program may be a useful addition to the training content in CT or CST treatments.

Present results showed that eleven followed participants maintained their improved recall scores for 3 months on the Rey–Osterrieth complex figure test. By contrast, we reported a patient who showed reduced recall scores after a 3-month interval (**Figure 6**, Case 3). Patients with relatively severe neurocognitive disorder (e.g., under 10 points in MMSE) might tend to show decline in cognitive benefits after non-intervention intervals. Participants with severe cognitive dysfunctions may need continuous treatment to maintain cognitive outcomes. Further study is needed to clarify the relationship between levels of neurocognitive dysfunction and retainment of cognitive outcomes.

We predicted that, as materials, natural flowers and leaves would be more attractive to participants than colored sticks and would encourage patients to participate continuously, similar to horticultural therapy. However, no significant differences were revealed between flowers and leaves versus colored sticks in attendance rates and cognitive outcomes. Performance times with flowers and leaves were significantly longer than with sticks. Real flowers and leaves’ sizes and forms differed each day, but the sticks’ size remained constant. Real flowers and leaves’ variety would make it difficult to arrange them and naturally increase time to completion, but that might be appropriate for further developing visuospatial ability.

In the SFA program, three-dimensional operations with materials were required, for example, in Pattern 1, one rose was placed straight up on the absorbent foam’s center, and other roses were placed at about 45° angles on its lateral sides (**Figure 2**); this is unique among visuospatial training tools including puzzles, peg-boards, and block designs (Mochizuki-Kawai et al., 2010). The present procedure would be difficult for patients with neurocognitive disorder, but we observed that almost all participants continued arranging materials without quitting and finished arrangements alone in the SFA intervention’s second half. The step-by-step procedures and impressions on the absorbent foam might have motivated patients and encouraged their continuous participation.

The time spent placing each piece of material did not decrease during each phase. Of two possible explanations for the lack of improvement in efficiency, first, we instructed patients to array materials in a correct and orderly way, but not quickly, so this likely influenced their performance time. Second, on the intervention’s second and third days, we asked patients to try a more complicated design than on the first day. Since the more complicated arrangement would require longer time, it might have canceled work efficiency. While we failed to detect significant time reduction, patients seemed to be acquiring skills (procedural memory) in creating their arrangements because they were able to finish by alone more complicated work on later treatment days.

Some differences appeared between the present study’s results and those of the previous study with schizophrenia patients. Present results showed improvement in scores of Rey–Osterrieth complex figure test, recall version, but not in the block-tapping task, whereas the previous study showed improvement in block-tapping task scores, but not in the Rey–Osterrieth complex figure test, recall version (Mochizuki-Kawai et al., 2010). Patients’ various diseases or degrees of neurocognitive dysfunction might have caused these differences.

Against our prediction, we did not detect significant improvement in Apathy Scale scores. However, nine SFA-treated patients maintained their positive mental state based on the Apathy Scale, while three of five SFA non-treated patients showed gradual psychic deterioration and became to be recognized as having apathy syndrome at the experiment’s final test. Perhaps the SFA intervention contributed to patients’ maintaining good mental health. There are some limitations in the present study. First, the SFA-treated patients continued to engage daily day-care activities during SFA interventions, so an interaction between the SFA program and daily activities would not be clear. Second, the present sample size was small especially with regard of the age, whereas we found stable benefits in SFA program treated patients who were from twenties to sixties. The cognitive benefits may be affected by the age and other related factors, such as brain reserve capacity, which designates the ability to maintain cognitive performance well in the face of age-related brain modifications and pathology (Franzmeier et al., 2017b; Gelfo et al., 2018). The brain reserve capacity is increased by the enriched early life experiences, for example, higher education, and induces greater brain connectivity during engaging memory task (Franzmeier et al., 2017a; Gelfo et al., 2018). The interactions between the SFA outcomes and aging or brain reserve capacity (e.g., education level) would be examined with larger sample in the future.

In the SFA program, patients took the flower and leaf arrangements home with them. We did not investigate the natural work’s influence in each home. The natural materials facilitate communication among participants’ family members and would ameliorate relationships between patients and caregivers, similar to CST interventions (Orrell et al., 2017). In the future, we plan to examine the SFA program’s effect on caregivers’ mental health and on relationships between patients and caregivers. Moreover, the SFA program may positively affect activities of daily living (ADL) levels because cognitive ability with visuospatial memory and recognition is associated with ADL level in neurocognitive disorders (Bieñkiewicz et al., 2015). We believe that the SFA program has much potential for improving cognitive and mental health in patients with brain damage and also for positively affecting both patients and family members’ QOL.

## Author Contributions

HM-K conceived and designed the study. HM-K, IK, and YY administered the cognitive intervention. HM-K, IK, and SM executed cognitive tests. HM-K analyzed the data and wrote the manuscript with the help of other members (SM, IK, and YY).

## Conflict of Interest Statement

IK is currently employed by company Sase Total Care Center Co., Ltd. The present study was executed independent of the company. The remaining authors declare that the research was conducted in the absence of any commercial or financial relationships that could be construed as a potential conflict of interest.
